# Prevalence and determinants of Italian physicians’ burnout in the “post-COVID-19” era

**DOI:** 10.1007/s00420-022-01929-6

**Published:** 2022-11-06

**Authors:** Elena Fiabane, Simona Margheritti, Edoardo Nicolò Aiello, Stefano Magnone, Massimo Miglioretti, Paola Gabanelli, Ines Giorgi

**Affiliations:** 1 Department of Physical and Rehabilitation Medicine of Genova Nervi Institute, Istituti Clinici Scientifici Maugeri IRCCS, Genoa, Italy; 2 Department of Psychology, University of Milano-Bicocca, Milan, Italy; 3 Bicocca Center for Applied Psychology (BiCApP), University of Milano-Bicocca, Milan, Italy; 4 PhD Program in Neuroscience, School of Medicine and Surgery, University of Milano-Bicocca, Milan, Italy; 5 ANAAO ASSOMED Lombardia Associazione Medici Dirigenti, Milan, Italy; 6 Psychology Unit of Pavia Institute, Istituti Clinici Scientifici Maugeri IRCCS, Pavia, Italy; 7 Dipartimento di Sanità Pubblica, Medicina Sperimentale e Forense, University of Pavia, Pavia, Italy

**Keywords:** Burnout, Physicians, Pandemic, COVID-19, Risk factors

## Abstract

**Purpose:**

Several studies investigated prevalence and determinants of physicians’ burnout during the peak of the COVID-19 pandemic, but only a few during the chronic phase of the pandemic. This study thus aimed to examine this topic referring to the “post-COVID-19 era”, defined as a chronic and likely-to-be endemic status quo.

**Methods:**

A cross-sectional, online survey (November 2021–January 2022) was addressed to physicians in Lombardia (Northern Italy). Besides socio-demographic and COVID-19-related data, measures of personal, work- and patient-related burnout (Copenhagen Burnout Inventory; CBI), depression (Patient Health Questionnaire-8), anxiety (General Anxiety Disorder-7), and self-efficacy (General Self-Efficacy Scale) were collected. Linear/generalized linear models were run to test associations/predictions of interest.

**Results:**

Among the 958 respondents, burnout symptoms were clinically significant in 18.5% of them. Predictive models showed that female sex (OR = 0.73, 95% CI 0.42–1.27), younger age (OR = 0.94, 95% CI 0.59–1.48), shorter job tenure (OR = 1.01, 95% CI 0.62–1.65), trainee status (OR = 1.41, 95% CI 1.16–7.10), higher PHQ-8 (OR = 1.260, 95% CI 1.16–1.37), and GAD-7 scores (OR = 1.19, 95% CI 1.10–1.30) increased the risk to suffer from clinical burnout. COVID-19-related variables were mostly not related/associated to burnout levels.

**Conclusion:**

In Italy, physicians’ burnout is moderately prevalent also in the chronic phase of the pandemic, with its determinants being more intrinsic than environmental. The development of effective interventions is needed to help physicians cope with the new challenges of their job.

**Supplementary Information:**

The online version contains supplementary material available at 10.1007/s00420-022-01929-6.

## Introduction

The COVID-19 pandemic has been demanding for healthcare institutions and professionals (Tan et al. 2020), as having prompted a redesign of organizational networks and having induced significant changes in practitioners’ personal and occupational lives.

Italy was one of the first and most detrimentally impacted countries during the outbreak in May 2020, with over 223,000 people infected and over 31,000 deaths (World Health Organization, 2020). In this context, healthcare workers faced an unexpected change in their job. The increase of workload, the uncontrollably increasing number of cases and deaths, the severe risk of contagion, the initial lack of personal protective equipment (PPE), the fear for their loved ones to be infected and the forced social separation from them had several consequences on the psychological health of healthcare workers (Fiabane et al., 2021; Giusti et al., 2020; Liu et al., 2020; Ran et al., 2020). In addition, the uncertainty, that characterized the emergency, left workers unprepared, leading them to feel confused, and consequently to perceive feelings of helplessness, alienation, and isolation (Giusti et al. 2020). In most of the cases, these reactions lead to several psychopathological outcomes such as anxiety, depression, insomnia, and stress (Bianchi et al. 2015; West et al. 2018; Zhang et al. 2019; Ran et al. 2020b; Baptista et al. 2021) increasing the risk of burnout syndrome (Sharifi et al. 2020).

Even 2 years after the onset of the pandemic, COVID-19 remains to have a considerable impact on healthcare workers’ well-being. Indeed, in January 2022, 220,532 new cases were registered, which represented the biggest daily increase in cases in Italy since the start of the pandemic (Statista, 2022). Nevertheless, the severity level of COVID-19 appeared to be less critical due to the 84% people vaccinated with full doses (Divino et al. 2022).

Traditional definitions of burnout syndrome include energy depletion or exhaustion, increasing mental distance from one’s employment or sentiments of negativism or cynicism about one’s own career, as well as lower professional efficacy (Schaufeli et al. 2009). Kristensen et al.’s (2005) burnout definition embraces various aspects of a worker’s life, such as personal, work-related, and client-related ones. Personal burnout is defined as physical, emotional, and cognitive fatigue and depletion, whereas work-related burnout refers to symptoms that individuals attribute to their specific work activity. Client-related burnout instead focuses on burnout symptoms related to workers’ feelings toward their target clients (e.g., patients for physicians, etc.).

Within several countries, high levels of burnout among healthcare professionals have been detected following the onset of the COVID-19 pandemic (Dinibutun 2020; Bradley and Chahar 2020; Sharifi et al. 2020; Conti et al. 2021; Dehon et al. 2021; Jalili et al. 2021; Fiabane et al. 2021; Thrush et al. 2021; Melnikow et al. 2022). However, most of these studies were conducted during the first year of the COVID-19 outbreak (Gorini et al. 2020; Andrijic et al. 2021; Jalili et al. 2021; Fiabane et al. 2021), thus exploring the psychological short-term impact and the professionals’ mental health status in an acutely stressful phase of response. However, even 2 years after the onset of the pandemic, COVID-19 still continues to have a significant impact on healthcare systems worldwide.

The scientific literature on previous outbreaks suggested that individuals who experience chronic stress and prolonged emotional burden are more likely to develop long-term psychological disturbances, including burnout, post-traumatic stress disorder (e.g., Maunder et al. 2006), and alcohol abuse (e.g., Wu et al. 2008).

Furthermore, to the best of our knowledge, only few studies have specifically explored the impact of COVID-19 on physicians’ burnout (Amanullah and Shankar 2020). Indeed, most studies focused on healthcare professionals in general (e.g., putting together nurses, physicians, and other healthcare workers) and, within such investigations, the medical profession was often under-represented, especially when compared to nurses (Andijic et al*.*, 2021; Fiabane et al., 2021; Jalili et al., 2021).

Burnout has been historically identified as a critical factor affecting physicians and their patients (Rotenstein et al. 2018). However, a need to specifically explore the impact of COVID-19 on physicians’ burnout has been recently highlighted (Amanullah and Shankar 2020). Indeed, although several risk factors for burnout in physicians have been identified within the early stages—including increased workload, fear of infection, isolation, change of clinical specialty, long working hours, prolonged use of PPEs (Giusti et al. 2020), being a frontline worker (Morgantini et al. 2020), working in emergency service, intensive care or public health (Cullen et al. 2020), studies are now needed to confirm these findings also in the post-acute phase. This, even more in the light of the availability of an anti-COVID-19 vaccine, the increased scientific knowledge on SARS-CoV-2, the decrease in deaths and hospitalization, the greater availability of PPEs and the reduction or removal of the social restrictions, which are important changes that healthcare workers have been only recently witnessed.

This epidemiological study thus aims to cross-sectionally capture the prevalence and determinants of burnout among a large sample of Italian physicians during the “post-COVID-19” era, herewith defined as the progressive transition from an acute, emergency state towards a chronic and likely-to-be endemic *status quo*, is still scarce (Zhou et al. 2022).

## Methods

### Participants

Italian healthcare graduated professionals (*N* = 18,516) working in public or private institutions in the Northern Italy were e-mailed a web-based questionnaire (Google Form) sent by ANAAO ASSOMED Lombardia Associazione Dirigenti Medici (Milan, Northern Italy). Data collection started in November, 2021 and ended in January, 2022. E-mails were forwarded three times, at a 14-day distance from one another, to achieve the highest possible response rate, by subtracting from each subsequent delivery those who had responded to the previous one. The e-mail was opened at least once by 3,832 people; 1,204 responses were obtained (response rate of 6.50%). Out of all respondents, 958 physicians practicing medical care were included in the final sample (Table 1) excluding health professionals (classified in supplementary Table 2) who were not physicians (e.g., pharmacists or veterinarians).

**Table 1:** Descriptive statistics of the sample

	*N* (%)
Sex	
Male	429 (44.8%)
Female	529 (55.2%)
Age	
< 30 years	13 (1.4%)
31–40 years	249 (26.0%)
41–50 years	277 (28.9%)
51–60 years	270 (28.2%)
> 60 years	149 (15.6%)
Job tenure	
< 5 years	72 (7.5%)
5–15 years	297 (35.4%)
16–25 years	250 (26.1%)
> 25 years	339 (31.0%)
Working area	
Emergency services	664(71.3%)
Not emergency services	267(28.7%)
Type of contract	
Open-ended contract	887 (92.6%)
Fixed-term contract	16 (1.7%)
Self-employed	31 (3.3%)
Trainee	24 (2.5%)

The study was approved by the Ethics Committee of the University of Milan-Bicocca (I.D.: RM-2021-451). Participants provided informed consent and data were treated according to current regulations.

### Materials

The web-based questionnaire was structured in three sections, aimed at collecting socio-demographic and occupational, COVID-19-related and psychological data, respectively (Table 2).

**Table 2:** Background data collected within the questionnaire

Demographics and employment data
1. Age
2. Sex
3. Job tenure
4. Specialization
5. Type of facility
6. Branch of activity
7. Actual scope of activity
8. Type of contract
Information related to the COVID-19 pandemic
9. Did you serve in the COVID-19 area?
10. Do you think the pandemic has impacted your well-being at work?*
11. Has anyone close to you had serious consequences due to SARS-CoV-2 infection?*
12. Has any of your colleagues had serious consequences due to SARS-CoV-2 infection*
Perceived burnout
13. According to the WHO definition of burnout^a^, do you think that you have been suffering from this syndrome?
14.If you answered “yes” to the previous question: do you think that you have suffered from burnout before or after the onset of the pandemic?
15. Have you ever feared that one day you might suffer from this syndrome?

*WHO *world health organization

*Questions addressed only to physicians who have served in COVID-19 unit

^a^At this point, a thorough definition of burnout according to the WHO was delivered to respondents

The third section included measures of burnout, anxiety, depression, and self-efficacy—the Copenhagen Burnout Inventory (CBI) (Avanzi et al. 2013; Aiello et al. 2022), the Generalized Anxiety Disorder-7 (GAD-7) (Spitzer et al. 2006), the Patient Health Questionnaire-8 (PHQ-8) (Kroenke et al*.*, 2009), and the Generalized Self-Efficacy Scale (GSE) (Chen et al. 2001), respectively.

The CBI by Aiello et al. (2022), adapted from Avanzi et al. (2013), is a physician-specific, self-report questionnaire assessing Personal, Work- and Client-related Burnout (PB; WB; CB). Its items range from 1 (“Never/almost never” for items 1–12 or “To a very low degree” for items 13–18) to 5 (“Always” for items 1–12 or “To a very high degree” for items 13–18), whereas its total score ranges from 18 to 90 (higher scores corresponding to higher burnout levels).

The GAD-7 and PHQ-8 self-reportedly screen for the presence of anxiety and depression symptoms within the last 2 weeks via Likert-scaled items where 0 corresponds to “Never” and 3 to “Almost every day”; higher GAD-7/PHQ-8 scores thus correspond to higher anxiety/depression levels.

The GSE comprises 10 Likert-scaled item self-reportedly assessing perceived self-efficacy by requiring subjects to express their agreement on quotes addressing their beliefs on their ability to act effectively in their live. Therefore, higher GSE scores correspond to higher levels of perceived self-efficacy.

### Statistical analysis

As, for all included outcomes, skewness and kurtosis values were ≤|1| and |3|, respectively (Kim 2013), univariate associations/predictions of interest addressing psychometric measures were tested via linear model analyses.

Prevalence estimates of clinical burnout were drawn by comparing CBI (Aiello et al. 2022) scores to respective cut-off values (> 10 for the GAD-7 and PHQ-8 and > 69 for the CBI).

To identify risk factors for clinical burnout within multivariate models, two separate multiple logistic regressions were run by addressing as the outcome a score ≤ 69 *vs.* > 69 on the CBI. The first model (M1) encompassed as predictors GAD-7, PHQ-8, and GSE scores along with age, sex, years of service, working state (trainee *vs.* self-employed *vs.* fixed term *vs.* open-ended), service in COVID-19 units (having *vs.* not having served), and specializations presumably linked to a greater exposure to SARS-CoV-2 infection (physicians serving in infectious disease, pulmonary, intensive care, and emergency units *vs.* others). The second model (M2) further encompassed, besides the full range of predictors entered into M1, those objective COVID-19-related variables available only for physicians having served in COVID-19 units, i.e., having or not witnessed to either a loved one or a colleague suffering from severe COVID-19.

SPSS 27 (IBM Corp., 2021) was adopted to analyze data. Significance level was set at *α* = 0.05 and multiple comparisons were Bonferroni-corrected whenever necessary.

## Results

### Descriptive findings

Table 1 shows socio-demographic, occupational and COVID-19-related data of participants.

71.6% of physicians stated that they had suffered from burnout, as defined by the World Health Organization. Out of these respondents, 66.2% postulated that they had suffered from burnout after the onset of the pandemic, whereas 33.8% before it. Furthermore, 59.5% of all respondents feared that they could suffer from it in the future, whereas 24.7% stated that they had never thought about it and 15.8% that they had never had such a fear. As for the 688 physicians who served in the COVID-19 area, only 1.6% declared that the pandemic had had no impact on their job, whereas the remaining 87.4% of respondents believed that the pandemic had affected it to some degree.

**Fig. 1:**
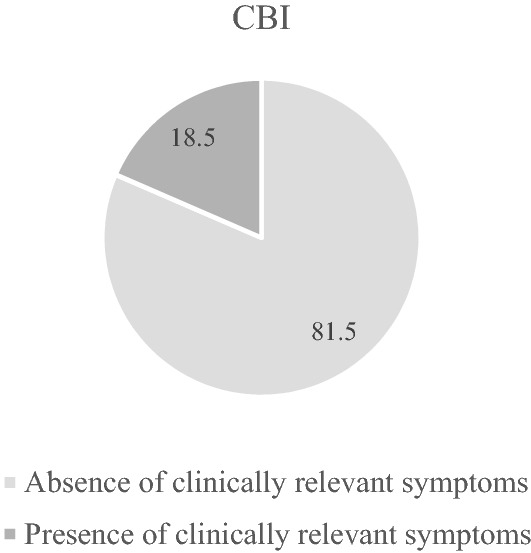
Percentage of clinically relevant symptoms in the whole sample (*N* = 958) as measured by the Copenhagen Burnout Inventory (CBI)

Descriptive statistics of psychometric measures are reported in Table 3. The prevalence of clinical burnout as detected by the CBI was 18.5% (Fig. 1). As to the association among psychometric measures, higher CBI scores proved to be positively related to both the GAD-7 and PHQ-8, whereas negatively to the GSE; the same association patterns emerged for the CBI sub-scales (WB, PB, and CB) (Supplementary Table 1).

**Table 3:** Descriptive statistics of psychometric measures

	M** ± **SD (range)
CBI-burnout	54.79 ± 15.15 (18–90)
PB—personal burnout	18.63 ± 5.46 (6–30)
WB—work burnout	21.75 ± 6.34(7–35)
CB—client burnout	14.41 ± 4.83(5–25)
PHQ-8-depression	8.79 ± 5.23 (0–24)
GAD-7-anxiety	8.15 ± 5.05 (0–21)
GSE-self-efficacy	28.44 ± 4.25 (10–40)

*CBI* Copenhagen burnout inventory, *PB *personal burnout, *WB* work burnout, *CB* client burnout, *PHQ-8* Patient Health Questionnaire-8, *GAD-7* general anxiety disorder-7, *GSE* General Self-Efficacy scale

### Association with psychometric measures

Associations between categorical variables and psychometric measures are summarized in Table 4

**Table 4:** Group comparison mean scores on dependent variables

Sex					
Measure	“Male” (*N* = 429)	“Female” (*N* = 529)			*F* statistics (*N* = 958)
CBI	52.13^a^ ± 15.19	56.95^b^ ± 14.79			24.63**
PB	17.39^a^ ± 5.44	19.63^b^ ± 5.28			41.61**
WB	20.65^a^ ± 6.37	22.64^b^ ± 6.18			23.70**
CB	14.08 ± 4.78	14.68 ± 4.86			3.70

Type of contract					
	“Open-ended” (*N* = 887)	“Fixed-term” (*N* = 16)	“Self-employed” (*N* = 31)	“Trainee” (*N* = 24)	*F* statistics (*N* = 958)
CBI	54.92 ± 15.27	52.00 ± 14.19	49.39 ± 13.75	58.88 ± 11.36	2.11
PB	18.62 ± 5.47	18.81 ± 5.91	17.29 ± 5.53	20.50 ± 4.51	1.57
WB	21.83 ± 6.39	20.75 ± 5.54	18.90 ± 5.67	23.42 ± 4.40	2.83*
CB	14.48 ± 4.91	12.44 ± 4.15	13.19 ± 3.37	14.96 ± 3.58	1.71

Having served in COVID-19 units-1					
	“Yes” (*N* = 688)	“No” (*N* = 270)			*F* statistics (*N* = 658)
CBI	54.90 ± 15.51	54.51 ± 14.21			0.13
PB	18.48 ± 5.59	18.99 ± 5.12			1.63
WB	21.85 ± 6.44	21.52 ± 6.08			0.52
CB	14.57 ± 4.93	14.01 ± 4.56			2.65

Having served in COVID-19 units-2					
	“I have never worked in a COVID-19 unit” (*N* = 270)	“Both in the first, second and third wave” (*N* = 471)	“Only in the second and third wave (from October 2020)” (*N* = 71)	“Only in the first wave, during the first lockdown (March and April 2020)” (*N* = 146)	*F* statistics (*N* = 958)
CBI	54.51 ± 14.21	54.83 ± 15.74	54.38 ± 16.38	55.40 ± 14.39	0.13
PB	18.99 ± 5.12	18.36^a^ ± 5.71	18.34^a^ ± 5.51	18.97 ± 5.23	1.03
WB	21.52 ± 6.08	21.78 ± 6.46	21.90 ± 6.98	22.03 ± 6.12	0.23
CB	14.01 ± 4.56	14.69 ± 4.93	14.14 ± 5.13	14.40 ± 4.83	1.22

Job role					
	“At high-risk for SARS-CoV-2 infection” (*N* = 187)	“Non-emergency role” (*N* = 771)			*F* statistics (*N* = 958)
CBI	53.34 ± 15.03	55.14 ± 15.17			2.13
PB	18.10 ± 5.55	18.75 ± 5.44			2.18
WB	21.28 ± 6.35	21.87 ± 6.33			1.31
CB	13.97 ± 4.89	14.52 ± 4.82			1.97

Perceived burnout					
	“No” (*N* = 272)	“Yes” (*N* = 686)			*F* statistics (*N* = 958)
CBI	42.76^a^ ± 12.62	59.56^b^ ± 13.32			319.04**
PB	14.57^a^ ± 4.77	20.23^b^ ± 4.86			266.44**
WB	16.67^a^ ± 5.26	23.77^b^ ± 5.56			326.91**
CB	11.51^a^ ± 4.18	15.56^b^ ± 4.58			160.13**

Perceived burnout: before *vs.* after COVID-19					
	“Before” (*N* = 248)	“After” (*N* = 485)			*F* statistics (*N* = 733)
CBI	57.57 ± 13.75	58.92 ± 13.80			1.58
PB	19.57 ± 5.10	19.97 ± 4.97			1.04
WB	22.99 ± 5.71	23.49 ± 5.75			1.25
CB	15.01 ± 4.74	15.46 ± 4.63			1.55

Perceived burnout: future					
	“No” (*N* = 151)	“Yes” (*N* = 570)	“I have never thought about it” (*N* = 237)		*F* statistics (*N* = 958)
CBI	51.75^a^ ± 17.10	55.82^b^ ± 14.46	54.26^a,b^ ± 15.25		4.53*
PB	17.25^a^ ± 6.16	18.95^b^ ± 5.24	18.72^b^ ± 5.41		5.88**
WB	20.74 ± 7.25	22.11 ± 6.04	21.54 ± 6.36		3.01
CB	13.76 ± 5.10	14.75 ± 4.66	14.00 ± 5.02		3.66*

Severe COVID-19 sequelae: colleagues					
	“No” (*N* = 246)	“Yes” (*N* = 442)			*F* statistics (*N* = 688)
CBI	53.12^a^ ± 15.13	55.89^b^ ± 15.65			5.06*
PB	17.91^a^ ± 5.42	18.80^a^ ± 5.66			3.92*
WB	21.13^a^ ± 6.24	22.24^a^ ± 6.52			4.71*
CB	14.07^a^ ± 4.84	14.85^a^ ± 4.96			3.93*

Severe COVID-19 sequelae: loved ones					
	“Yes” (*N* = 261)	“No” (*N* = 427)			*F* statistics (*N* = 688)
CBI	56.56^a^ ± 15.44	53.89^b^ ± 15.49			4.83*
PB	19.27 ± 5.48	18.01 ^b^ ± 5.61			8.37**
WB	22.51 ± 6.39	21.44 ^b^ ± 6.44			4.44*
CB	14.79 ± 5.05	14.44 ± 4.85			0.80

^a,b^Mean scores on the same dependent variables with diverse apical letters differed significantly across groups (*p < .05, **p < .001.), as a result of the Bonferroni correction

When compared to males, female participants systematically scored higher on the CBI. The type of job contract was associated only with WB. No differences in CBI scores were found between those who served or not in COVID-19 units. The same result yielded when further differentiating those who served continuously in such units since the first wave from those who did it only in the first or second/third wave. Moreover, no differences were found between specialists presumed to be more highly exposed to SARS-CoV-2 infection when compared to other ones. At *α*_adjusted_ = 0.025, CBI scores were inversely related to both age (− 0.16 ≤ *r*(958) ≤ − 0.13; *p* < 0.006) and years of service (− 0.17 ≤ *r*(958) ≤ − 0.14; *p* < 0.006).

Participants who subjectively stated that they had likely suffered from burnout, when compared to those who did not, reported higher CBI scores; however, within the former group, no differences were found between those who postulated that they had suffered from it before *vs.* after the onset of the pandemic. Participants who stated that they feared that they could suffer from burnout in the future reported higher CBI scores, with not differences being detected against those stating that they had never had such a concern.

Participants who had served in COVID-19 units and whose loved ones (relatives and/or close friends) had experienced severe sequelae of SARS-CoV-2 infection showed higher CBI scores than those who had not experienced such a situation. Similarly, having had colleagues with severe COVID-19 was associated with higher score on the CBI in physicians who had served in the COVID-19 area. Moreover, in such a physician group, a greater perceived impact of the pandemic on their work well-being was found to be associated with higher CBI score (*r*(688) = 0.37; *p* < 0.001).

### Predictive model of clinical burnout

Tables 5, 6 show results of multiple logistic regression analysis of different variables to detect their impact on the development of clinical burnout.

**Table 5:** Model 1: predictors of a CBI score above *vs.* below the cut-off (whole sample)

						95% confidence interval	
Predictor	Estimate	SE	Z	*p*	Odds ratio	Lower	Upper
PHQ-8	0.19401	0.0334	5.8129	< .00001	1.214	1.137	1.2962
GAD-7	0.19849	0.0343	5.7838	< .00001	1.220	1.140	1.3044
GSE	− 0.03840	0.0288	− 1.3341	0.18216	0.962	0.910	1.0182
Job tenure (year classes)	− 0.05436	0.2139	− 0.2541	0.79938	0.947	0.623	1.4403
Age (year classes)	− 0.00937	0.2002	− 0.0468	0.96270	0.991	0.669	1.4668
Type of job contract							
Open-ended *vs.* fixed term	3.37856	1.2725	2.6550	0.00793	29.328	2.422	355.1819
Freelancer *vs.* fixed term	1.62607	1.6195	1.0041	0.31535	5.084	0.213	121.5362
Trainee *vs.* fixed term	2.98096	1.4295	2.0854	0.03704	19.707	1.196	324.6230
Having served in COVID-19 units							
Yes *vs.* no	− 0.41073	0.2658	− 1.5453	0.12228	0.663	0.394	1.1165
At-risk health sectors							
At-risk *vs.* not-at-risk	− 0.40756	0.3100	− 1.3145	0.18867	0.665	0.362	1.2215
Sex							
Female *vs. *male	− 0.35710	0.2404	− 1.4852	0.13749	0.700	0.437	1.1209

*PHQ-8* Patient Health Questionnaire-8, *GAD-7* Generalized Anxiety Disorder-7, *GSE* General Self-Efficacy scale

Coefficients *b *represent the log odds of a CBI score above the cut-off (> 69) *vs*. below (≤ 69)

**Table 6:** Model 2: predictors of a CBI score above *vs.* below the cut-off (physicians having served in COVID-19 unit)

						95% CI	
Predictor	*b*	*SE*	*z*	*p*	*OR*	Lower	Upper
PHQ-8	0.2313	0.0427	5.4207	< .00001	1.260	1.1591	1.37007
GAD-7	0.1768	0.0424	4.1668	0.00003	1.193	1.0982	1.29697
GSE	− 0.0282	0.0336	− 0.8389	0.40153	0.972	0.9103	1.03836
Job tenure (year classes)	0.0121	0.2485	0.0486	0.96120	1.012	0.6219	1.64727
Age (year classes)	− 0.0658	0.2347	− 0.2806	0.77904	0.936	0.5911	1.48301
At-risk health sectors							
At-risk *vs.* not-at-risk	− 0.6425	0.3391	− 1.8944	0.05817	0.526	0.2706	1.02249
Severe sequelae of COVID-19: loved ones							
Yes *vs.* no	− 0.2041	0.2834	− 0.7203	0.47135	0.815	0.4678	1.42097
Severe sequelae of COVID-19: colleagues							
Yes *vs.* no	− 0.3803	0.2980	− 1.2762	0.20188	0.684	0.3812	1.22600
Perceived impact of the pandemic*	0.5482	0.1519	3.6101	0.00031	1.730	1.2848	2.32989
Sex							
Female *vs.* male	− 0.3190	0.2842	− 1.1222	0.26177	0.727	0.4164	1.26886
Type of job contract							
Open-ended *vs.* fixed term	3.2991	1.5999	2.0621	0.03920	27.087	1.1775	623.12478
Freelancer *vs.* fixed term	1.4119	1.9345	0.7299	0.46547	4.104	0.0926	181.88549
Trainee *vs.* fixed term	1.6974	1.8366	0.9242	0.35540	5.460	0.1492	199.76320

*PHQ-8* Patient Health Questionnaire-8, *GAD-7* Generalized Anxiety Disorder-7, *GSE *General Self-Efficacy scale

Coefficients *b* represent the log odds of a CBI score above the cut-off (> 69) *vs*. below (≤ 69). *Higher scores corresponding to a higher perceives impact

Within M1, which addressed the whole sample, GAD-7 (χ^2^(1) = 35.68; *p* < 0.001) and PHQ-8 scores (χ^2^(1) = 36.37.; *p* < 0.001) predicted a higher probability of clinical burnout (GAD-7: *OR* = 1.22, 95% CI [1.14, 1.30]; PHQ-8: *OR* = 1.21, 95% CI [1.14, 1.30]). Moreover, an effect of the type of job contract was detected (χ^2^(3) = 14.21; *p* = 0.002), with fixed-termed physicians being at lower risk of clinical burnout when compared to both open-ended (*OR* = 29.33, 95% CI [2.42, 355.18]) ones and trainees (*OR* = 19.71, 95% CI [1.2, 324.62]). Remaining predictors yielded no significance. M2, which included only those physicians that have served in COVID-19 units, yielded similar results, with GAD-7 and PHQ-8 predicting a higher probability of an above-cut-off CBI score (both *p*s < 0.001), as well as with type of job contract (χ^2^(3) = 11.46; *p* = 0.009) affecting the occurrences *vs.* absence of clinical burnout, with open-ended physicians being at higher risk when compared to fixed-term ones (*OR* = 27.09, CI 95% [1.18, 623.12]).

## Discussion

This study aimed to investigate the prevalence and determinants of burnout among Italian physicians during the latest and current phases of the COVID-19 pandemic. To the best of our knowledge, the present research was the first conducted on a large sample of physicians exploring the chronic and long-term impact of COVID-19 on their burnout levels.

Psychometric measurements yielded an estimated prevalence of clinical burnout of 18.5% in this population. Despite this value seemingly being more conservative when compared to previous Italian studies during peak pandemic periods (e.g., Conti et al., 2021), it has to be noted that the standardized measure herewith adopted, i.e., the CBI, as well as its cut-off, are physician-specific (Aiello et al. 2022). Moreover, the normative CBI value addressed within this study conveys diagnostic information, as referring to burnout levels that deserve clinical attention (Aiello et al. 2022). Taken together, such considerations suggest that the prevalence of burnout yielded from this survey validly captures the actual cross-sectional picture, not incurring in overestimation biases. A recent review (Rotenstein et al. 2018) on the prevalence of burnout among physicians before the COVID-19 pandemic showed substantial variability in prevalence estimates of burnout ranging from 0% to 80.5% based on its definitions, assessment methods, and study quality. Rotenstein and colleagues underlined the lack of clear consensus among the 182 studies included in this review, and suggested the use of measurement tools such as CBI that avoids conceptual problems and is freely available (Rotenstein et al, 2018).

Additionally, in this study, it is worth noting that a consistency was detected between objective burnout levels and subjective reports of having suffered from it/fearing to suffer from it in the future—with participants delivering such statements actually having higher CBI scores. Specifically, those who suspected that they had experienced burnout reported higher levels in all the three CBI sub-scales (PB, WB, and CB). At the same time, those who feared that they could suffer from burnout in the future reported higher PB and CB scores. Therefore, physicians were seemingly able to correctly recognize their own fatigue and depletion symptoms, as well as the possibility that their psychological status may negatively affect their relationship with patients.

In line with the previous studies, female physicians were found to experience higher levels of general burnout (West et al. 2018; Baptista et al. 2021; Jalili et al. 2021), PB and WB, as compared to males. No differences were detected in terms of CB, this indicating that males and females did not differ in burnout levels based on their role in caring for their patients. Some of these sex differences could be explained by sociocultural factors such as gender norms, which have been identified as critical factors in understanding the work-life interface. Gender norms imposed by society have placed significant stresses on women, posing unique obstacles to balancing work and family commitments (Linzer and Harwood 2018; Baptista et al. 2021; Singe et al. 2022). Balancing work and family duties became more challenging during the advent of the COVID-19 pandemic (Halley et al. 2021; Rabinowitz and Rabinowitz 2021; Ayar et al. 2022) when the increasing workload and the fear of infecting their families’ members severely impacted on the psychological well-being of women physicians.

In line with previous studies, the present results also showed that younger physicians (Chou et al. 2014; West et al. 2018; Barello et al. 2020; Baptista et al. 2021) and those with shorter job tenure (West et al. 2018; Baptista et al. 2021) experienced higher levels of burnout compared to others.

In addition, participants who reported that their work well-being was affected by the pandemic showed higher levels of burnout—especially as to PB. Having had loved ones who experienced severe COVID-19 sequelae also was associated with higher burnout levels, also as to PB, WB, and CB dimensions. These results suggest that not simply the pandemic per se, but its effect on people closed to physicians, and thus their personal experience, might determine higher levels of stress in the latter.

Findings from predictive models highlighted the crucial role of subjective factors in determining physicians’ burnout levels. In this respect, results revealed higher anxiety and depression levels, along with an open-ended contract or trainee status, as risk factors for clinical burnout. The notion of anxiety and depression being associated with and predictive of burnout is unsurprising (Rossi et al. 2020; Pappa et al. 2020). Findings on the type of job contract are in line with the literature only as far as trainees are concerned since they have less stable employment status and the lowest seniority of service (Wielers et al. 2022). At the same time, trainees may be the most affected by factors such as inadequate training, job insecurity, and financial stress, which were found to be among the main causes of anxiety, depression, and burnout symptoms at the time of Covid-19 (Lulli et al. 2021). By contrast, the finding of open-ended physicians being at higher risk for burnout when compared to fixed-term ones is counterintuitive and, as likely being biased by the fact that the first contract type was the most represented (887 physicians out of 958), poorly interpretable.

It is, however, striking that neither having served in COVID-19 units nor serving within high-risk healthcare sectors (e.g., infectious disease unit) were found as risk factors for burnout. These findings are in contrast with most studies conducted during the peak phases of the pandemic, when being a frontline worker with close contact with infected patients was associated with higher level of psychological disturbances (i.e., anxiety, depression, stress) (West et al. 2018; Amanullah and Shankar 2020; Hossain et al. 2021). Our result can be attributed to the reorganization of the at-risk health sector, which currently entails safety procedures by practitioners, with likely reduction in stress levels.

It is well known that burnout in doctors was present long before the pandemic (Rotenstein et al, 2018); these results could show that after the increased burnout during the acute phase of outbreak (e.g., Fiabane et al. 2021) it has come back to the "regular" levels. This also in the light of the fact that COVID-19-related variables in this study were mostly not related/associated to burnout, suggesting that in this chronic phase of COVID-19 pandemic it is not the virus per se to determine the high prevalence of burnout.

Therefore, our study showed that the “post-pandemic era” is characterized by different risk factors, since the context of emergency has changed and factors influencing acute stress in the early stages of the pandemic may significantly differ from those affecting the current one. For example, a recent study found that COVID-19 pandemic-related chronic stress has profound impacts on the long-term mental health of the general population, suggesting that the acute and chronic effects of the pandemic are influenced by different factors (Qui et al., 2021). If the early phase of the pandemic was characterized by an acute stress reaction, needed to cope with several contextual stressors, the current, chronic phase could be considered as a second response of adaptation, which includes the acceptance of what has happened and the attempts of rebuilding individual and professional lives. The response to stress in this phase significantly depends on individual risk factors and resources and could be thus less influenced by contextual factors. This can help explain the key role of depression and anxiety in influencing burnout in this study, as well as the lack of significance of work-related factors that, by contrast, have been identified as highly relevant in the previous, peak phases.

Although these findings are of particular relevance in the actual healthcare scenario, this study has some limitations. First, because of the cross-sectional nature of our study, causal relationships cannot be inferred; therefore, a longitudinal design would be necessary to explore the causality of these relationships. Second, this research is based on the exclusive use of online self-reported measures and thus suffers the limitations of such a methodology.

Despite these limitations, this study has the merit to be the first one to address Italian physicians’ burnout during the “post-COVID-19 era”, exploring specific risk factors that characterize this chronic stage. Furthermore, it should be emphasized that such an investigation offered a contemporary picture of burnout prevalence in a large sample of Italian medical professionals.

## Practical implication

Our findings identified relevant risk factors for physicians’ burnout during the “post-COVID-19” era. It follows that there is a need to develop individual-level interventions designed to promote resilience and the use of adaptive coping strategies. Stress management programs that range from relaxation to cognitive-behavioral and patient-centered therapy could help achieve this goal (Romani and Ashkar 2014; Amanullah and Shankar 2020). However, a recent review (Lulli et al. 2021) aimed to explore psychosocial factors contributing to occupational stress during the current pandemic suggested the protective role of organizational factors, such as support from colleagues and organizations, good workload management, appropriate training and home-work balance.

Therefore, combining individual and organizational interventions should be the most effective strategy in preventing burnout. Thus, multidisciplinary actions that include regular work stress/burnout assessment procedures (Argentero et al. 2010), changes in the environment (e.g., reducing poor work environment, excessive job demands, and poor work-life balance) along with stress management programs could be more effective. Organizational interventions based on training, improved teamwork, workflow, and organizational restructuring are helpful to reduce burnout among younger and trainees’ physicians (Zhou et al. 2020). Instead, promoting better work-life balance and increase control in their workplace should prevent women’s burnout (McMurray et al. 2000; Amanullah and Shankar 2020) helping in managing duties and workload.

## Conclusion

The present research showed that clinical burnout is moderately prevalent among Italian physicians during the chronic phase of the COVID-19 pandemic. Both individual and environmental, work-related factors contribute to burnout that physicians experience. These include depression, anxiety, younger age, female sex, and working as a trainee. Risk factors for burnout that have emerged during the peak stages of pandemic were not detected in this study (i.e., working in COVID-19 units or in high-risk healthcare sectors), suggesting that the determinants of physicians’ burnout changed from the former stages to the current, chronic one, in which individual resilience and personal coping strategies play a pivotal role. Such data can inform institutions and mental health practitioners devoted to implement burnout preventive programs in healthcare professionals.

## Supplementary Information

Below is the link to the electronic supplementary material.

Supplementary file1 (DOCX 18 KB)

## Data Availability

Data sets associated with the present study are available upon request of interested researchers.
