# Supplementary material for: Prevalence and determinants of Italian physicians’ burnout in the “post-COVID-19” era
Source: Int Arch Occup Environ Health. 2022 Nov 6;96(3):377–87. doi: 10.1007/s00420-022-01929-6 (PMC9638242; doi:10.1007/s00420-022-01929-6)
Supplement: Supplementary file 1 — Supplementary file1 (DOCX 18 KB) [file 420_2022_1929_MOESM1_ESM.docx]

**Supplementary Table 1.** Pearson’s coefficients among psychometric measures.

|  | **CBI** | **PB** | **WB** | **CB** | **GSE** | **PHQ-8** | **GAD-7** |
| --- | --- | --- | --- | --- | --- | --- | --- |
| **CBI** | -- |  |  |  |  |  |  |
| **PB** | .914* | -- |  |  |  |  |  |
| **WB** | .958* | .867* | -- |  |  |  |  |
| **CB** | .839* | .599* | .728* | -- |  |  |  |
| **GSE** | -.388* | -381* | -.394^*^ | -270* | -- |  |  |
| **PHQ-8** | .757* | .773* | .743* | .527* | -.383* | -- |  |
| **GAD-7** | .724* | .743* | .705* | .507* | -.404* | .825* | -- |

**Notes.** *Significant coefficients at threshold α_adjusted_=.017. CBI=Copenhagen Burnout Inventory; PHQ-8=Patient Health Questionnaire-8; GAD-7=General Anxiety Disorder-7; GSE=General Self Efficacy Scale.

**Supplementary Table 2.** Descriptive statistics about physicians’ area of work

|  | **%** |
| --- | --- |
| **Health-care specialization** |  |
| Allergology and clinical immunology | 0.3 |
| Other | 2.6 |
| Anesthesia and Resuscitation | 11.3 |
| Audiology and Phoniatrics | 0.1 |
| Clinical Biochemistry | 0.1 |
| Cardiac Surgery | 0.7 |
| Cardiology | 6.8 |
| General surgery | 8.0 |
| Maxillofacial surgery | 0.5 |
| Pediatric surgery | 0.9 |
| Plastic and reconstructive surgery | 0.2 |
| Thoracic surgery | 0.3 |
| Vascular surgery | 1.1 |
| Dermatology and venereology | 0.6 |
| Hospital medical direction | 0.1 |
| Hematology | 1.4 |
| Endocrinology | 1.5 |
| Pharmacology and clinical toxicology | 0.5 |
| Gastroenterology | 1.6 |
| Medical Genetics | 0.1 |
| Geriatrics | 1.9 |
| Gynecology and obstetrics | 5.5 |
| Hygiene, epidemiology, and public health | 0.2 |
| Respiratory diseases | 2.6 |
| Infectious Diseases | 3.2 |
| Metabolic Diseases and Diabetology | 0.2 |
| Emergency Medicine | 2.4 |
| Occupational Medicine | 1.0 |
| Physical Medicine and Rehabilitation | 1.6 |
| Internal Medicine | 9.6 |
| Legal Medicine | 0.2 |
| Nuclear Medicine | 0.1 |
| Transfusional Medicine | 0.7 |
| Microbiology and Virology | 0.1 |
| Nephrology | 3.3 |
| Neonatology | 0.7 |
| Neurosurgery | 1.0 |
| Neurology | 2.8 |
| Child Neuropsychiatry | 2.3 |
| Ophthalmology | 1.3 |
| Oncology | 2.5 |
| Orthopedics and Traumatology | 4.3 |
| Otolaryngology | 1.3 |
| Pediatrics | 2.9 |
| Psychiatry | 4.9 |
| Radio diagnostics | 0.5 |
| Radiotherapy | 0.4 |
| Rheumatology | 0.8 |
| Food science and dietetics | 0.5 |
| Urology | 2.1s |
